# Mobile health applications for enhancing mental health access and outcomes among adolescents and young people living with HIV: a systematic review

**DOI:** 10.1186/s12982-026-01998-9

**Published:** 2026-05-06

**Authors:** Carlos Muleya, Rosemary N. Likwa, Jacqueline J. Folotiya, Jeremia Banda, Peter J. Chipimo, Patricia Sakala, Melvin Salati, Chikoloma Nakazwe, Linah K. Mwango, Caitlin Baumhart, Cassidy W. Claassen, Naeem Dalal, Loyd B. Mulenga

**Affiliations:** 1 University of Zambia, Lusaka, Zambia; 2 World Health Organization, Lusaka, Zambia; 3 CAZACHA Business Solutions Limited, Lusaka, Zambia; 4 Young African Statisticians Association, Lusaka, Zambia; 5 Ciheb Zambia, Lusaka, Zambia; 6 Institute of Human Virology, University of Maryland School of Medicine, Baltimore, MD USA; 7 Zambia National Public Health Institute, Lusaka, Zambia; 8 Ministry of Health, Lusaka, Zambia

**Keywords:** Mental health, Access, Mobile applications, Adolescents and young people, HIV

## Abstract

**Background:**

Mental health (MH) disorders remain a major global public health concern, disproportionately affecting adolescents and young people living with HIV (AYPLHIV). Limited access to MH services, stigma, and a shortage of trained professionals hinder effective care, particularly in low-resource settings. Mobile health (mHealth) applications have emerged as accessible and scalable tools for improving MH support and reducing disparities in service delivery. This systematic review assessed the effectiveness of mHealth applications in improving access to MH services and outcomes among AYPLHIV and comparable populations globally.

**Methods:**

A systematic search was conducted across PubMed, PsycINFO, Web of Science, and the Cochrane Library for studies published between January 2020 and March 2025. Eligible studies evaluated mobile or app-based interventions designed to enhance MH access, engagement, or outcomes. Systematic reviews, meta-analyses, SMS-only interventions, and non-empirical studies were excluded. Two independent reviewers (CM and PS) screened articles, extracted data, and assessed study quality using the Joanna Briggs Institute (JBI) Critical Appraisal Tools. Data were synthesized narratively following the Preferred Reporting Items for Systematic Reviews and Meta-Analyses (PRISMA) 2020 framework.

**Results:**

Of 265 records identified, 16 empirical studies met the inclusion criteria. The studies covered Africa (Ethiopia, Kenya), Asia (Indonesia, Vietnam, Iran, Malaysia), Europe (Spain, Poland, United Kingdom), North America (USA), and Oceania (Australia), and employed randomized controlled trials, mixed-methods, pre–post, and cross-sectional designs. Across studies, mobile interventions consistently improved user engagement, accessibility, and MH outcomes, including significant reductions in depression, anxiety, and stress symptoms (*p* < 0.05). Peer-based models delivered via WhatsApp demonstrated high feasibility and acceptability among AYPLHIV, while cognitive behavioral therapy (CBT) and mindfulness-based apps such as IntelliCare and Headspace produced outcomes comparable to traditional care in several settings. Key facilitators included personalization, app usability, and human or AI-enabled feedback (e.g., chatbot support), while barriers included privacy concerns, connectivity constraints, and low digital literacy.

**Conclusions:**

mHealth applications substantially improve access to and delivery of MH services for AYPLHIV and related groups. Integration of these tools into existing health systems, combined with culturally tailored design, ethical AI use, and sustainability strategies, can strengthen MH care globally. Future research should address long-term impact, cost-effectiveness, and equity in implementation.

**Supplementary Information:**

The online version contains supplementary material available at 10.1186/s12982-026-01998-9.

## Introduction

Mental health (MH) disorders are a significant public health concern globally, affecting approximately one in four individuals across all age groups [1]. According to the World Health Organization (WHO), mental disorders account for a growing proportion of the global disease burden, with depression being among the leading causes of disability worldwide and suicide remaining the fourth leading cause of death among individuals aged 15–29 years [2]. Despite global recognition of the importance of MH, access to MH services remains extremely limited, particularly in low- and middle-income countries (LMICs), where more than 75% of people with mental disorders receive no formal treatment [3]. Structural challenges, including a shortage of trained professionals, weak health infrastructure, stigma, and inadequate financing, continue to widen the treatment gap. In sub-Saharan Africa, particularly in Zambia, access to MH care is constrained by systemic and socioeconomic barriers. Zambia’s MH workforce remains critically low, with fewer than one psychiatrist per 500,000 people, and only approximately 20% of health facilities are equipped to provide MH services [4]. Stigma surrounding mental illness and cultural misconceptions often discourages individuals from seeking care, while existing facilities are concentrated in urban centers, leaving rural communities largely underserved [5]. Adolescents and young people are disproportionately affected, facing a double burden of limited access and heightened psychosocial vulnerability.

Among adolescents and young people living with HIV (AYPLHIV), these challenges are amplified by the complex intersection of HIV-related stigma, discrimination, poverty, and social isolation, which contribute to elevated rates of depression, anxiety, and substance use [6, 7]. Studies indicate that AYPLHIV are significantly more likely to experience MH disorders than their HIV-negative peers are, which is often linked to the stress of living with a chronic condition, fear of disclosure, and medication adherence pressures [8]. Moreover, substance use, including alcohol and drug misuse, has emerged as both a coping mechanism and a risk factor that exacerbates MH problems and increases the likelihood of poor adherence to antiretroviral therapy (ART) [9]. This multidimensional vulnerability underscores the urgent need for scalable, youth-friendly, and confidential interventions to promote MH and adherence among this population.

Recognizing these challenges, the WHO and other global health stakeholders have called for the integration of MH services into HIV care and treatment programs to ensure holistic, person-centered care [10]. However, this recommendation remains difficult to implement in many LMICs, where the scarcity of specialized professionals and limited funding for MH services persist [11]. To overcome these gaps, innovative and cost-effective digital approaches are increasingly being explored as viable alternatives for expanding access to MH care.

Mobile health (mHealth) applications have emerged as promising solutions to bridge these access gaps by leveraging widespread mobile phone ownership, even in low-resource settings. Globally, more than 6.8 billion people own or use mobile phones, including a growing number of adolescents and young adults [12]. In sub-Saharan Africa, the penetration of smartphones and social media platforms has created new opportunities to remotely deliver MH interventions. mHealth applications provide convenient, private, and stigma-free platforms for AYPLHIV to access counseling, psychoeducation, peer support, and symptom tracking [13]. Different types of mobile interventions have been developed and deployed to support MH among young people. These include instant messaging platforms such as WhatsApp and Facebook Messenger, which facilitate real-time communication with counselors and peer groups; SMS-based interventions, which deliver reminders, psychoeducational messages, and motivational content; and dedicated smartphone apps available on the Google Play store or Apple App Store, which offer interactive features such as mood monitoring, guided relaxation, cognitive‒behavioral exercises, and connections to virtual care providers [14].

Despite their growing popularity and demonstrated benefits, the empirical evidence on the effectiveness of these mHealth applications in improving access to MH services among AYPLHIV remains fragmented. Variations in study design, population focus, and implementation context have limited the comparability of results across settings. Therefore, this systematic review aimed at synthesizing and critically appraising the evidence on how mobile applications and app-based platforms influence access to MH services and MH-related outcomes among AYPLHIV and comparable adolescent and young adult populations globally. By consolidating evidence across diverse contexts, this review seeks to inform the design, implementation, and evaluation of scalable digital MH strategies integrated within HIV and youth health services.

## Methods

A comprehensive search strategy was developed using medical subject headings (MeSH) and relevant keywords, including terms related to AYPLHIV aged approximately 10–24 years, mobile applications, mental health, and access to services. The five-year period (2020–2025) was selected to prioritize the most recent and policy-relevant evidence, recognizing the rapid evolution of mobile application ecosystems, accelerated uptake of digital health during the COVID-19 period, and continuous changes in smartphone access, connectivity, and app features. Search strings were adapted for each database (PubMed, PsycINFO, Web of Science, and the Cochrane Library). A representative PubMed search strategy is provided in Supplementary File 1.

**Fig. 1:**
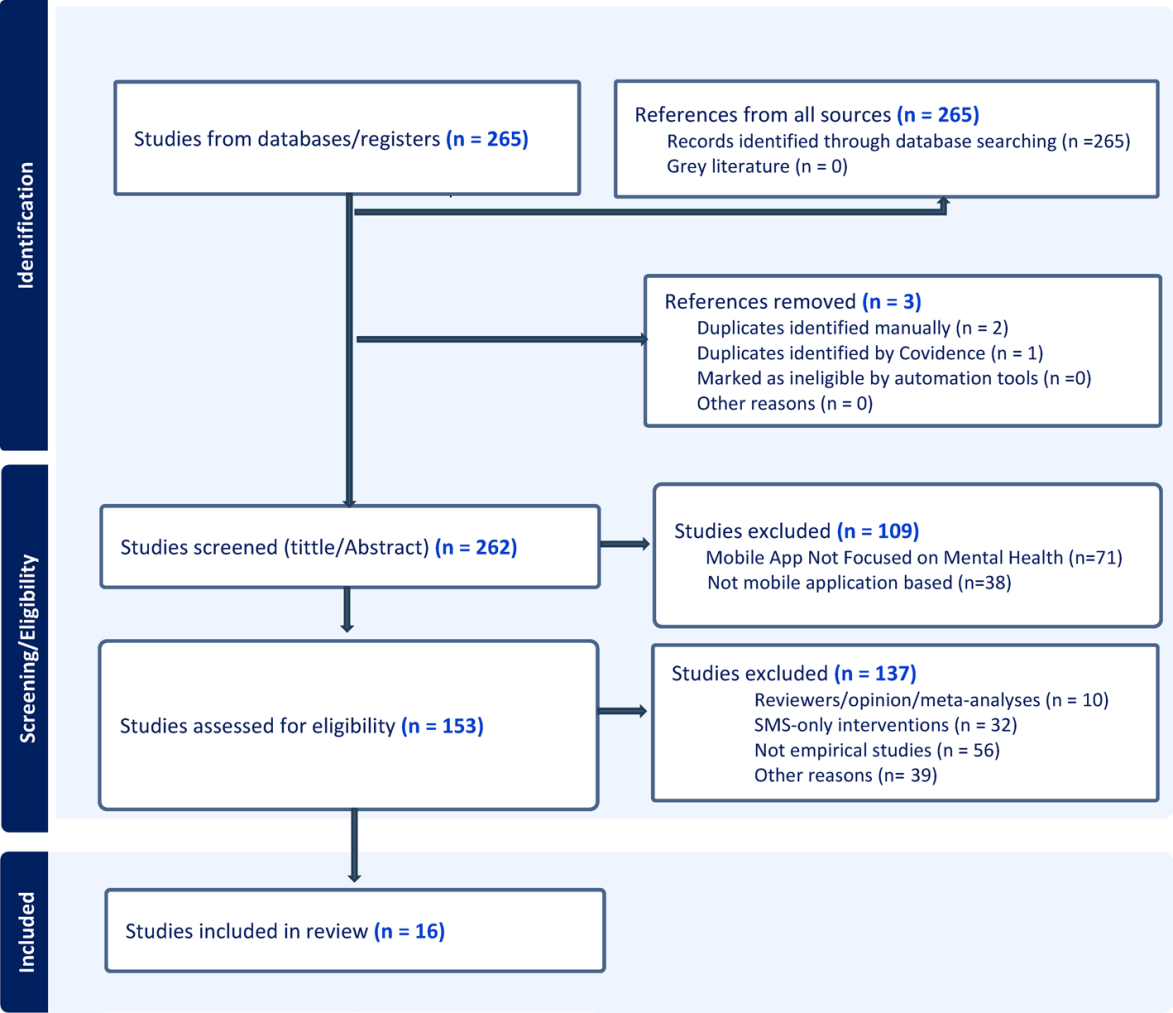
Preferred Reporting Items for Systematic Reviews and Meta-Analyses (PRISMA) 2020 flow diagram Source: Generated

Studies were included if they (a) evaluated the use of mHealth applications or app-based platforms to improve access to or delivery of MH services among AYPLHIV or comparable populations; (b) were empirical studies (quantitative, qualitative, or mixed methods) published in English between 2020 and 2025; and (c) reported at least one measurable outcome related to access, engagement, or MH improvement. Studies were excluded if they (a) focused solely on SMS-based or web-only interventions without an mHealth application component; (b) were systematic reviews, meta-analyses, or opinion papers; (c) were nonempirical (protocols, commentaries, or theoretical papers); or (d) fell outside the date or language limits. The findings were synthesized narratively following the Preferred Reporting Items for Systematic Reviews and Meta-Analyses (PRISMA) 2020 guidelines [15]. The detailed selection process is summarized in Fig. 1 (PRISMA flow diagram), which illustrates the identification, screening, and inclusion of the 16 studies summarized in Table 1 (data extraction table).

Two independent reviewers (Carlos Muleya and Patricia Sakala) used a standardized data extraction form to capture key information, including author(s), year of publication, study aim, country or setting, study design, sample characteristics, outcomes measured, key findings, and quality assessment. The extracted data were cross-checked for accuracy, and discrepancies were resolved through discussion or consultation with a third reviewer (Chikoloma Nakazwe).

The methodological rigor of each included study was appraised via the Joanna Briggs Institute (JBI) Critical Appraisal Tools, selecting the checklist appropriate for each study design (e.g., RCT, quasi-experimental, or cross-sectional). The criteria evaluated included clarity of objectives, appropriateness of methodology, reliability of measurement tools, and completeness of outcome reporting. Each study was rated as high, moderate, or low quality. Overall, the evidence base comprised 16 high- or moderate-quality studies, as summarized in Table 1.

**Table 1:** Characteristics of included studies (*n* = 16) and quality appraisal Source: Generated

S/*N*	Author(s)	Year	Country/setting	Aim	Study design	Sample size	Outcomes	Key findings	Quality appraisal
1	Guracho YD, et al.	2024	Ethiopia	Identify determinants of MH app use among youth.	Cross-sectional study.	423	Access and utilization of MH apps; determinants of use.	Approximately 50% of youth expressed strong interest in MH apps; about 21% were current users. Uptake was higher among urban and more educated participants.	High quality
2	Saberi P, et al.	2021	USA	Assess MH and substance use effects on ART adherence in youth with HIV.	Mixed-methods study.	68	ART adherence; MH and substance use patterns; acceptability of digital supports.	Substance use and depressive symptoms were linked with lower adherence; participants endorsed the value of technology-mediated support to address stigma and access barriers.	High quality
3	Chory A, et al.	2021	Kenya	Evaluate WhatsApp for counseling and peer support for adolescents with HIV.	Mixed-methods study.	50	Feasibility and acceptability; engagement; social support; adherence-related outcomes.	WhatsApp-based support was feasible and acceptable; participants reported improved social connectedness and psychosocial support, with perceived reductions in stigma.	High quality
4	Rahayu TB, et al.	2024	Indonesia	Evaluate a mobile app for adolescent MH monitoring.	Pre–post experimental study.	50	Depression and anxiety symptoms; help-seeking and monitoring behavior.	App use supported symptom monitoring and reduced self-reported distress over the intervention period.	High quality
5	Ariyanti S, et al.	2024	Indonesia	Evaluate effectiveness of a MH app in reducing depression among adolescents.	Pre–post experimental study.	60	Depression symptoms; app engagement.	The intervention reduced depressive symptom severity and was acceptable to adolescents.	High quality
6	Nam LH, et al.	2025	Vietnam	Evaluate app-based therapy for anxiety and depression in adolescents/young adults.	Mixed-methods study.	95	Depression and anxiety outcomes; user acceptability; feasibility.	Significant reductions in anxiety and depression symptoms were reported, supported by high acceptability of app-based therapy.	High quality
7	Rickard NS, et al.	2022	Australia	Assess the quality of MH apps using the APA evaluation model.	App quality assessment study.	90 apps	App usability, evidence base, privacy/security, and clinical foundation.	Apps varied widely in quality; many lacked robust evidence or clear privacy protections, underscoring the need for structured evaluation before implementation.	Moderate quality
8	Graham AK, et al.	2020	USA	Evaluate IntelliCare platform efficacy for depression/anxiety in primary care.	Randomized controlled trial.	146	Depression and anxiety symptom reduction; engagement/adherence.	IntelliCare produced higher engagement and clinically meaningful improvements in depression and anxiety compared with control conditions.	High quality
9	Edge D, et al.	2024	United Kingdom	Evaluate MyMoodCoach to support mental well-being in young people.	Randomized controlled trial.	236	Mental well-being; rumination; internalizing symptoms; feasibility.	The intervention improved rumination and well-being, with signals of benefit for internalizing symptoms among youth.	High quality
10	Fiol-DeRoque MA, De la Torre-Luque A, et al.	2021	Spain	Evaluate PsyCovidApp for mental health support during COVID-19.	Randomized controlled trial.	497	Stress, anxiety, and depression symptoms; app adherence.	PsyCovidApp improved stress management and reduced anxiety symptoms relative to control, particularly among participants already receiving therapy.	Moderate quality
11	Kirk U, et al.	2023	USA	Evaluate Headspace mindfulness to reduce subjective and physiological stress reactivity.	Randomized controlled trial.	163	Stress and anxiety symptoms; stress reactivity.	Headspace users reported reductions in perceived stress with improvements in stress reactivity during the intervention.	High quality
12	Ngure K, et al.	2023	Kenya	Assess feasibility of Vijana-SMART WhatsApp peer support for adolescents living with HIV.	Feasibility study (pilot).	55	Feasibility and acceptability; engagement; psychosocial support.	High feasibility and acceptability were reported, with strong engagement and perceived improvements in peer support and connectedness.	High quality
13	Karkosz S, et al.	2024	Poland	Evaluate Fido chatbot for young adults with subclinical symptoms.	Randomized controlled trial.	81	Loneliness, depression and anxiety symptoms; app engagement.	High-frequency users showed reductions in loneliness and improvements in emotional well-being; engagement was associated with better outcomes.	Moderate quality
14	Soltani Z, et al.	2024	Iran	Evaluate the Yara app for major depression.	Randomized controlled trial.	64	Depression and anxiety symptoms; sleep quality; adherence.	Participants using Yara reported significant reductions in depressive symptoms and improvements in sleep quality compared with control.	High quality
15	Tan S, et al.	2023	Malaysia	Evaluate MoodMission as an add-on to treatment-as-usual.	Randomized controlled trial.	48	Depression and anxiety outcomes; acceptability.	MoodMission as an adjunct improved depressive and anxiety symptoms compared with treatment-as-usual alone, with good user acceptability.	High quality
16	Zawadzki MJ, et al.	2025	USA	Evaluate Headspace mindfulness app among employees using ecological momentary assessment.	Randomized controlled trial using ecological momentary assessment.	138	Stress, anxiety, and resilience; real-time engagement measures.	The intervention reduced stress and improved coping in real-world assessments; sustained engagement was supported by app reminders and guided content.	Moderate quality

Given the heterogeneity of study designs and outcome measures, a narrative synthesis approach was employed. The findings were grouped by (a) user engagement and access outcomes, (b) mental health outcomes (e.g., anxiety, depression, and stigma reduction), and (c) barriers to and facilitators of implementation. This structure ensured direct comparability with the results section and alignment with the PRISMA framework.

This systematic review did not require ethics approval, as it did not involve the collection of primary data from human participants. However, the protocol, which included the systematic review, received ethical clearance from the local Institutional Review Board (IRB), National Health Research Authority (NHRA), and Ministry of Health at all levels (Head Quarters, Provincial, District and Facility levels).

## Results

### Study selection and characteristics

A total of 265 records were retrieved from the database search. After removal of duplicates (*n* = 3) and screening of titles and abstracts, 153 full-text articles were assessed for eligibility. Of these, 137 were excluded for reasons including: reviews and meta-analyses (*n* = 10), SMS-only interventions (*n* = 32), studies that were not empirical (*n* = 56), and other reasons (*n* = 39). After eligibility screening, 16 unique studies published between 2020 and 2025 were included for synthesis.

**Fig. 2:**
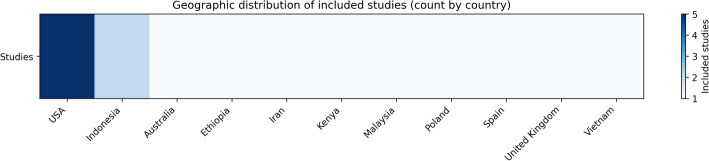
Geographic distribution of included studies (count by country). Source: Generated.

The included studies were conducted in diverse geographic regions, including Africa (Ethiopia, Kenya), Asia (Indonesia, Vietnam, Iran, Malaysia), Europe (Spain, Poland, United Kingdom), North America (USA), and Oceania (Australia) as shown in Fig. 2.

The studies used a variety of designs, including—randomized controlled trials (*n* = 8), mixed-methods (*n* = 4), pre–post experimental (*n* = 3), and cross-sectional (*n* = 1)—and collectively enrolled over 2000 participants. All studies examined the use of mobile applications or app-based platforms to improve MH access, engagement, or psychosocial outcomes among AYPLHIV and related adolescent and young adult populations as shown in Table 2.

**Table 2:** Summary of intervention modalities across included studies (*n* = 16) Source: Generated

Intervention modality	Examples	No. of studies	Key outcomes assessed
Peer-support social platforms (WhatsApp)	Vijana-SMART; WhatsApp counseling/peer groups	2	Feasibility, acceptability, social support, stigma, adherence
CBT/skills-based apps and therapy programs	IntelliCare; MyMoodCoach; MoodMission; Yara; app-based therapy	7	Depression/anxiety symptoms, well-being, sleep, engagement
Mindfulness apps	Headspace (RCT and EMA)	2	Stress/anxiety symptoms, coping, engagement
AI-enabled chatbot tools	Fido	1	Loneliness and emotional well-being; engagement
Psychoeducation/stress support apps	PsyCovidApp	1	Stress and anxiety outcomes; adherence
App quality/evidence evaluation	APA evaluation model assessment	1	Usability, evidence base, privacy/security
Determinant/acceptability studies	Youth app-use determinants; digital support preferences	2	Interest in apps, uptake and barriers/facilitators

### User engagement and access to care

High levels of user engagement were reported across most interventions. African studies using WhatsApp-based platforms (Ngure et al., Chory et al.) achieved participation rates above 85%, demonstrating the feasibility of integrating peer-support models into mobile ecosystems for AYPLHIV [16, 17]. In Ethiopia, Guracho et al. observed that approximately 50% of youth expressed strong interest in MH apps and about 21% were active users, with higher uptake among urban and more educated participants [18].

In Asia, applications such as Yara (Iran) and MoodMission (Malaysia) achieved high user satisfaction, while Headspace and IntelliCare demonstrated sustained adherence throughout intervention periods. Features that promoted sustained engagement included reminders, goal tracking, and human or AI-enabled feedback [19–23].

### Mental health outcomes

All 16 studies reported measurable improvements in at least one psychological outcome, including depression, anxiety, or perceived stress.

### Depression reduction

Ten studies reported significant decreases in depressive symptoms following app use. For example, IntelliCare users demonstrated two- to threefold higher recovery rates compared to control groups [19], and the MyMoodCoach digital program showed improvements in internalizing symptoms and well-being among youth [24].

### Anxiety and stress

Apps such as Headspace and Yara significantly reduced anxiety and improved sleep quality (*p* < 0.001) [20, 21, 23]. PsyCovidApp improved stress management, particularly among participants already engaged in therapy [25].

### Adherence and stigma reduction

In Kenya, WhatsApp-based peer-support interventions facilitated improved ART adherence, reduced stigma, and increased social connectedness among AYPLHIV [16, 17].

### Other outcomes

Fido reduced perceived loneliness among high-frequency users [26], and MyMoodCoach improved rumination and well-being in adolescents and young adults [24].

Overall, 75% of the studies included reported statistically significant improvements (*p* < 0.05) in depression or anxiety outcomes, demonstrating consistent benefits across settings and mHealth modalities.

### Barriers to access and implementation challenges

Common barriers included technical limitations (unstable internet connectivity, phone sharing), privacy concerns, and limited digital literacy, particularly in rural or low-resource contexts. The participants in several African and Asian studies [16, 17, 27, 28] expressed hesitancy to discuss personal MH information digitally because of confidentiality concerns. Younger users and those in urban settings reported greater comfort and sustained use of mHealth applications.

At the system level, challenges included inadequate integration of mHealth tools into existing health services and a lack of clinician or peer follow-up. Several RCTs [22, 25] noted diminished effects when mHealth applications were used without structured human support, suggesting that hybrid digital–human models may yield better outcomes.

### Facilitators and enabling features

Facilitators identified across studies included personalization of content, in-app reminders, AI-assisted feedback, and social connectivity. Chatbots such as Fido were effective in providing immediate stigma-free interaction and psychoeducation [26]. Peer-based designs, as used in WhatsApp interventions among AYPLHIV, were especially valued for their sense of community and mutual encouragement [16, 17].

High usability scores (≥ 80/100) in PsyCovidApp and IntelliCare [19, 25] emphasize the importance of intuitive interface design. Across regions, participants valued 24/7 access, privacy, and anonymity, which were repeatedly cited as motivators for sustained use.

Overall, the evidence demonstrates that mHealth applications—either standalone cognitive behavioral therapy (CBT)-based apps, mindfulness platforms, or moderated social media interventions—significantly increase access to MH support, symptom management, and psychosocial well-being [16–31]. While implementation challenges remain, particularly regarding equity and integration into existing systems, the results confirm the scalability and adaptability of mHealth technologies as effective digital health interventions for AYPLHIV and the broader population.

## Discussion

This systematic review synthesized evidence from sixteen empirical studies published between 2020 and 2025 evaluating how mHealth applications improve access to MH services among the AYPLHIV and related populations. The findings confirm that mobile and app-based interventions – delivered through smartphone applications, chatbots, or moderated social media platforms – enhance psychological outcomes, engagement, and accessibility of care across diverse settings. Collectively, these tools offer CBT, mindfulness, psychoeducation, and peer-support functions that mitigate both MH burdens and access barriers to traditional in-person care.

African-based studies using WhatsApp and Facebook platforms [16, 17] demonstrated exceptional feasibility and acceptability, achieving participation rates exceeding 85% and promoting psychosocial support among AYPLHIV. The participants reported improved self-efficacy, social connectedness, and treatment adherence, with parallel reductions in stigma and emotional distress. A study conducted in Ethiopia [18] revealed that more than half of young respondents expressed strong interest in mobile MH apps, revealing an unmet demand for technology-mediated care.

Evidence from Asia, Europe, and North America further supports the efficacy of CBT and mindfulness-based apps such as IntelliCare [19], Headspace [23], [24], Fido [26], and PsyCovidApp [25]. These programs produced clinically meaningful reductions in depression, anxiety, and stress within intervention periods ranging from two to eight weeks, often matching the effects of traditional therapy. The Yara app [21] and MoodMission [22] confirmed similar benefits in low- and middle-income contexts, showing significant improvements in anxiety, sleep, and adherence outcomes. User engagement and retention were consistently highest in interventions incorporating real-time feedback, peer interaction, or personalized guidance.

These results are consistent with prior meta-analyses demonstrating that mobile MH interventions can generate moderate-to-large effects on depressive and anxiety symptoms when grounded in CBT or mindfulness frameworks [13, 14]. This review extends existing knowledge by highlighting recent evidence from diverse settings, including feasibility and acceptability findings for WhatsApp-enabled peer support among AYPLHIV in Kenya and determinants of MH app uptake among youth in Ethiopia [16–18]. Together, these studies underscore the potential of culturally adapted, low-barrier digital platforms to complement formal health-care systems, particularly where specialist MH services are limited.

Technological innovation, particularly artificial intelligence (AI)-driven conversational agents, has also emerged as a significant theme. Chatbots such as and Fido [24, 26] provided automated CBT and self-reflection guidance with minimal clinician input, demonstrating scalability and feasibility for continuous MH support. Mindfulness and psychoeducational platforms (Headspace, Yara) [21, 23] enhance emotional regulation and resilience, whereas usability studies (PsyCovidApp, IntelliCare) [19, 25] highlight the importance of personalization and intuitive design. Across studies, usability ratings averaged above 80% confirmed that the user experience is a critical determinant of sustained engagement.

Within HIV care, digital MH platforms can be integrated into differentiated service delivery models as low-intensity support options, with referral pathways for higher-intensity services when needed. For AYPLHIV, WhatsApp-based peer support and structured CBT or mindfulness apps may offer scalable complements to clinic-based counseling, particularly when combined with human facilitation or clinician oversight to sustain engagement and ensure safety.

Cultural and linguistic adaptation proved to be equally important. Locally designed or translated applications such as *Yara* [21] in Iran and *MoodMission* [22] in Malaysia achieved excellent adherence, reinforcing that contextual relevance and inclusivity should be central to mHealth application development. Policymakers and implementers in LMICs are therefore encouraged to prioritize participatory codesign, ensuring that applications reflect local norms, digital literacy levels, and user needs.

### Age-tailored design considerations

Developers and implementers should differentiate adolescents from older young adults when designing and deploying MH apps. Age-tailored approaches may include simplified content, school- and family-sensitive scheduling, enhanced confidentiality features, and optional caregiver engagement pathways that preserve adolescent autonomy. Such tailoring is particularly important for AYPLHIV, where stigma and disclosure concerns can shape digital help-seeking behaviors.

Moreover, the studies highlight the strategic opportunity to leverage AI and data analytics for adaptive, real-time feedback. Predictive algorithms embedded within MH apps could identify users at risk of relapse or disengagement, enabling proactive support. Nevertheless, privacy, security, and ethical oversight must remain foundational to maintain trust and protect sensitive user data.

Limitations include the heterogeneity of interventions (e.g., social media peer support, CBT skills training apps, mindfulness apps, chatbot-based tools) and variability in user-interface design and feature sets, which may influence engagement and outcome effects across studies. Although the evidence base is strong, several methodological limitations should be acknowledged. Most trials were short in duration (2–12 weeks) and involved relatively small sample sizes, limiting the assessment of long-term outcomes. The majority relied on self-reported psychological measures rather than clinician-rated assessments, which may introduce reporting bias. Regional representation also remains uneven, with limited data from Latin America and the Middle East. Furthermore, heterogeneity in intervention types, outcome measures, and theoretical frameworks complicates direct comparisons across studies. Finally, few studies have addressed gender, socioeconomic, or digital literacy disparities, leaving important equity considerations insufficiently explored.

Overall, the reviewed studies provide robust and convergent evidence that mHealth applications significantly increase access to and delivery of MH services across global contexts. By offering scalable, user-centered, and context-sensitive interventions, mobile technologies reduce treatment gaps and empower individuals to manage their psychological well-being proactively. As digital health ecosystems mature, mobile MH applications—supported by human facilitation and ethical AI design—represent a critical innovation for achieving equitable and sustainable MH service delivery worldwide.

## Conclusion and recommendations

### Conclusion

This systematic review demonstrated that mHealth application technology has emerged as a powerful and adaptable tool for improving access to MH services, particularly among AYPLHIV in both high- and low-resource settings. Across the 16 studies published between 2020 and 2025, mHealth applications – ranging from CBT-based interventions and mindfulness platforms to peer support and AI-assisted chatbots – consistently enhanced user engagement, adherence, and MH outcomes, including reductions in depression, anxiety, and perceived stress.

In Africa, the use of widely available social media platforms such as WhatsApp and Facebook provided cost-effective means for delivering psychosocial support, reducing stigma, and improving ART adherence among AYPLHIV. In Asian and Western contexts, CBT and mindfulness-based apps such as Headspace, and IntelliCare demonstrated strong evidence for improving emotional regulation, well-being, and accessibility of MH support outside of clinical environments. Collectively, these findings highlight that mobile MH tools can bridge significant gaps by offering scalable, private, and flexible support options for young people who may otherwise face stigma or logistical barriers to MH care.

### Recommendations

#### Integration into health systems

Governments and implementing partners should integrate mobile MH applications into existing HIV and primary health-care programs. This will promote continuity of care and ensure that digital tools complement, rather than replace, human-delivered MH services.

#### Cultural and linguistic adaptation

Developers should co-design mHealth applications with end-users to ensure contextual relevance, cultural appropriateness, and language accessibility. Locally adapted content has been shown to improve acceptability and sustained engagement.

#### Hybrid and human-supported models

The evidence supports combining mobile tools with counselor or peer facilitation to enhance engagement and long-term adherence. Hybrid delivery models should be prioritized to balance automation with personalized human support.

#### Ethical AI and data security

As AI-enabled MH tools become more common, robust frameworks must be established to ensure data privacy, transparency, and ethical use of predictive analytics. Protecting sensitive health information is critical to user trust and long-term adoption.

#### Capacity building and digital literacy

Training programs for health care workers and AYPLHIV should emphasize digital literacy to enable the effective use of mHealth technologies and reduce disparities in access among rural or low-resource populations.

#### Sustainability and evaluation

Policymakers and funders should invest in longitudinal studies that assess the cost effectiveness, scalability, and sustainability of mobile interventions, especially in LMIC contexts. Continued evaluation ensures that applications remain responsive to evolving user needs.

#### Future research priorities

Future studies should prioritize longer follow-up periods, cost-effectiveness analyses, and implementation outcomes (reach, fidelity, scalability, and equity) across both low- and high-resource settings. Comparative effectiveness designs that assess which app features (e.g., peer support, clinician oversight, AI-enabled personalization) drive sustained engagement will strengthen the evidence base for policy and program decision-making.

#### Leveraging artificial intelligence

Future development should incorporate AI-driven chatbots, sentiment analysis, and predictive algorithms to provide personalized feedback and early identification of users at risk for psychological distress while maintaining ethical oversight and transparency.

In conclusion, mobile MH applications present an unprecedented opportunity to expand equitable access to MH care globally. By integrating these tools within existing health systems, enhancing cultural adaptability, and embedding AI responsibly, policymakers and researchers can leverage technology to reduce barriers, close access and treatment gaps, and promote long-term well-being among AYPLHIV.

## Supplementary Information

Supplementary Material 1.

## Data Availability

No datasets were generated or analysed during the current study.
