# Supplementary material for: Mobile health applications for enhancing mental health access and outcomes among adolescents and young people living with HIV: a systematic review
Source: Discov Public Health. 2026 May 6;23(1):673. doi: 10.1186/s12982-026-01998-9 (PMC13149599; doi:10.1186/s12982-026-01998-9)
Supplement: Supplementary file 1 — Supplementary Material 1. [file 12982_2026_1998_MOESM1_ESM.docx]

# Supplementary File 1. Representative PubMed Search Strategy

This supplementary file provides a representative search strategy used for PubMed. The search syntax was adapted for PsycINFO, Web of Science, and the Cochrane Library, using equivalent controlled vocabulary and keyword fields as appropriate.

**Search limits used:**

- Language: English
- Publication dates: 01 January 2020 to 31 March 2025
- Population: adolescents and young people (approximately 10–24 years) and related adolescent/young adult populations relevant to AYPLHIV

**PubMed search (example):**

(("Mobile Applications"[MeSH] OR "mHealth"[Title/Abstract] OR "mobile health"[Title/Abstract] OR "mobile app"[Title/Abstract] OR "mobile application"[Title/Abstract] OR smartphone*[Title/Abstract] OR app-based[Title/Abstract]))

AND

(("Mental Health"[MeSH] OR "Mental Disorders"[MeSH] OR "Depression"[MeSH] OR "Anxiety"[MeSH] OR "psychological"[Title/Abstract] OR "depression"[Title/Abstract] OR "anxiety"[Title/Abstract] OR "stress"[Title/Abstract] OR "psychosocial"[Title/Abstract]))

AND

(("Adolescent"[MeSH] OR "Young Adult"[MeSH] OR adolescent*[Title/Abstract] OR youth[Title/Abstract] OR "young people"[Title/Abstract] OR "young adult"[Title/Abstract]))

AND

(("HIV"[MeSH] OR HIV[Title/Abstract] OR "human immunodeficiency virus"[Title/Abstract] OR "AIDS"[Title/Abstract] OR "antiretroviral"[Title/Abstract]) OR (AYPLHIV[Title/Abstract] OR "young people living with HIV"[Title/Abstract]))

AND

((access*[Title/Abstract] OR uptake[Title/Abstract] OR engagement[Title/Abstract] OR utilization[Title/Abstract] OR "service delivery"[Title/Abstract] OR "health services accessibility"[MeSH]))

Note: Depending on database functionality, the “HIV” concept may be implemented as (a) a required concept for AYPLHIV-focused searches, and/or (b) a supplementary concept to capture studies in related adolescent and young adult populations relevant to service access and delivery. The final study set is determined by the eligibility criteria and screening process reported in the manuscript.

## Database yield reporting

Total records retrieved across all databases (after exporting results and prior to de-duplication): n = 265. If required by the journal, database-specific retrieval counts can be reported from the original export logs or reference manager database.
